# Comprehensive Analysis of a ceRNA Network Identifies lncR-C3orf35 Associated with Poor Prognosis in Osteosarcoma

**DOI:** 10.1155/2020/3178037

**Published:** 2020-09-21

**Authors:** Yi Shi, Jianhua Ren, Ze Zhuang, Wenhui Zhang, Zhe Wang, Yuangao Liu, Jinze Li, Tangzhao Liang, Ronghan He, Kun Wang

**Affiliations:** ^1^Department of Joint and Trauma Surgery, Third Affiliated Hospital of Sun Yat-sen University, China; ^2^Department of Orthopedics, First Affiliated Hospital of Anhui Medical University, China

## Abstract

Osteosarcoma is a highly malignant bone cancer which primarily occurs in children and young adults. Increasing evidence indicates that long noncoding RNAs (lncRNAs), which function as competing endogenous RNAs (ceRNAs) that sponge microRNAs (miRNAs) and messenger RNAs (mRNAs), play a pivotal role in the pathogenesis and progression of cancers. The regulatory mechanisms of lncRNA-mediated ceRNAs in osteosarcoma have not been fully elucidated. In this study, we identified differentially expressed lncRNAs, miRNAs, and mRNAs in osteosarcoma based on RNA microarray profiles in the Gene Expression Omnibus (GEO) database. A ceRNA network was constructed utilizing bioinformatic tools. Kaplan-Meier survival analysis showed that lncR-C3orf35 and HMGB1 were associated with poor prognosis of osteosarcoma patients. Furthermore, results of Gene Set Enrichment Analysis (GSEA) suggested that lncR-C3orf35 may be involved in cellular invasion, the Toll-like receptor signaling pathway, and immune cell infiltration in the tumor microenvironment. Further analysis showed that patients with osteosarcoma metastasis expressed higher levels of lncR-C3orf35 and HMGB1 compared to metastasis-free patients. Moreover, the metastasis-free survival rate of the high lncR-C3orf35/HMGB1 expression group was significantly lower than that of the low expression group. The ESTIMATE algorithm was used to calculate the immune score and stromal scores for each sample. High lncR-C3orf35 and HMGB1 levels were correlated with low immune scores. ImmuCellAI analysis revealed that a low proportion of macrophage infiltration was associated with high lncR-C3orf35 and HMGB1 expression. The differential expression of lncR-C3orf35, miR-142-3p, and HMGB1 was further verified by quantitative real-time PCR. This study indicates that lncR-C3orf35 could be considered as a novel potential biomarker and therapeutic target of osteosarcoma, which may contribute to a better understanding of ceRNA regulatory mechanisms.

## 1. Introduction

Osteosarcoma, the most common bone malignancy, is a major cause of cancer-associated mortality in children and adolescents [1]. Osteosarcoma primarily affects the terminus of long bones, such as proximal tibias and distal femurs. Approximately 5 new osteosarcoma cases occur per million people under the age of 20 each year in America [2]. The incidence of osteosarcoma has increased by 0.3% per year over the last decades [3]. Although surgery and chemotherapeutic regimens have recently achieved notable progress in the treatment of osteosarcoma, the prognosis of osteosarcoma is still unsatisfactory. For patients exhibiting osteosarcoma metastasis at diagnosis, the 5-year survival rate is less than 30% [4]. The mechanisms underlying osteosarcoma carcinogenesis and progression have not been fully elucidated, and novel biomarkers such as therapeutic targets and predictors of prognosis are needed to be investigated.

Long noncoding RNAs (lncRNAs), of more than 200 nucleotides in length, are a major class of noncoding RNAs. Initially, lncRNAs were regarded as “junk genes” during transcription due to the absence of protein-coding capacity [5]. Recently, studies have shown that lncRNAs are involved in various biological processes, including gene transcription, chromatin modification, and epigenetic regulation [6]. Salmena et al. [7] determined that lncRNAs functioned as competing endogenous RNAs (ceRNAs). The ceRNAs act as sponges for target microRNAs (miRNAs) and regulate the miRNA-induced gene silencing. miRNAs are a class of short noncoding RNAs which contain 18 to 25 nucleotides. miRNAs can bind to base-complementary mRNAs, leading to inhibition of translation or degradation of mRNAs [8]. Moreover, aberrant expression of lncRNAs has been reported to be involved in the occurrence and development of osteosarcoma [9]. Increased lncRNAs including MALAT1 [10], TUG1 [11], and 91H [12] in osteosarcoma act as oncogenes to promote tumor proliferation and invasion. Conversely, decreased expressions of loc285194 [13] and MEG3 [14] have been reported to act as tumor suppressors in osteosarcoma. However, the majority of lncRNAs have not yet been functionally characterized in the pathogenesis of osteosarcoma and warrant further investigation.

In this study, osteosarcoma data were collected from the Gene Expression Omnibus (GEO) (http://www.ncbi.nlm.nih.gov/geo/), which includes 41 osteosarcoma samples and 8 normal controls. A ceRNA network with 14 lncRNAs, 9 miRNAs, and 58 mRNAs was obtained based on bioinformatic prediction analysis. Kaplan-Meier survival analysis of an independent dataset identified lncR-C3orf35 and HMGB1 as genes associated with survival. In addition, the potential biological activity and clinical features of lncR-C3orf35/HMGB1 were analyzed. The expression of the lncR-C3orf35/miR142-3p/HMGB1 axis was validated by quantitative real-time PCR (qRT-PCR) in osteosarcoma cells. Our study was aimed at analyzing the epigenetic mechanisms of osteosarcoma prognosis and providing support for lncRNAs as potential biomarkers.

## 2. Materials and Methods

### 2.1. Collection of RNA Expression Profiles

RNA expression profiles of osteosarcoma were collected from GEO. lncRNA and mRNA microarray datasets were deposited by Sadikovic et al. [15] and Fritsche-Guenther et al. [16] with accession numbers GSE12865 and GSE14593, respectively (Table 1). The GSE12865 dataset comprised 12 osteosarcoma tissue samples and 2 normal tissue samples, while the GSE14359 dataset included 10 osteosarcoma and 2 normal tissue samples. The GSE28423 dataset was deposited by Namløs et al. [17] and included miRNA expression profiles of 19 osteosarcoma cell lines and 4 normal osteoblast cell lines. The 3 datasets were used to screen differentially expressed RNAs in osteosarcoma. We downloaded the GSE21257 [18] dataset which includes 53 osteosarcoma patients with mRNA and lncRNA expression profiles and clinical features for further analysis.

### 2.2. Identification of Differentially Expressed RNAs

We directly downloaded RNA expression series-matrix profiles, which had been processed by the original authors. The names of lncRNAs, miRNAs, and mRNAs in the microarrays were annotated using the corresponding GEO platforms. Expression measurements of multiple probe sets mapping to the same genes were averaged, and the probe sets mapping to multiple genes were removed. The *p* < 0.05 and logfold change (FC) > 1 were used as the cut-off criteria. Differentially expressed RNAs with statistical significance between osteosarcoma and normal controls in each dataset were identified using the limma R package. Differentially expressed (DE) RNAs in the GSE12865 and GSE14359 datasets were merged. The miRcode [19] lncRNA annotation was used to screen lncRNA genes. Hence, using this approach, we could identify DE mRNAs (DEmRNAs), lncRNAs (DElncRNAs), and miRNAs (DEmiRNAs).

### 2.3. Establishment of the ceRNA Regulatory Network

lncRNA-miRNA interactions for DElncRNAs and DEmiRNAs were predicted using miRcode [19]. miRNA-mRNA regulatory pairs among DEmiRNAs and DEmRNAs were obtained using miRTarBase [20], TargetScan [21], and miRDB [22]. Overlapping regulatory pairs were identified by Venn diagrams, which were then used to construct the lncRNA-miRNA-mRNA network based on the union of DElncRNA-DEmiRNA pairs and DEmiRNA-DEmRNA pairs. Cytoscape v3.7.1 [23] software was used to visualize the ceRNA work.

### 2.4. Survival Analysis

Fifty-three samples with RNA expression profiles and survival information in GSE21257 were used for the Kaplan-Meier (KM) survival analysis [24]. All lncRNAs and mRNAs in the ceRNA network were included. A *p* value < 0.05 was set as the cut-off criterion. Then, the survival-associated lncRNA-mRNA pairs were selected and visualized with KM survival curves using the survival R package.

### 2.5. Regression Analysis of the lncR-C3orf35/HMGB1 Pair

Regression analysis of the expression of survival-associated lncRNA and mRNA was completed by Spearman's correlation analysis and was visualized by the ggpubr R package. *p* < 0.05 and *r* > 0.3 were used as the selection criteria.

### 2.6. Gene Set Enrichment Analysis for lncR-C3orf35

To identify the key biological process and signaling pathway affected by lncRNA, we classified samples into high and low expression lncR-C3orf35 groups. Gene Ontology (GO) biological process and Kyoto Encyclopedia of Genes and Genomes (KEGG) pathway enrichment analyses were conducted using Gene Set Enrichment Analysis (GSEA) v4.0.2 (https://software.broadinstitute.org/gsea/downloads.jsp) to analyze the mRNA expression profile of GSE21257.

### 2.7. Clinical Feature Analysis for lncR-C3orf35/HMGB1

The GSE21257 dataset contains demographic and metastasis information for 53 osteosarcoma patients. We compared the lncR-C3orf35 and HMGB1 expression levels of the metastasis and nonmetastasis groups using unpaired *t*-test. The samples were divided into the high expression group and low expression group based on median lncR-C3orf35 or HMGB1 levels. The high expression group consisted of 26 samples, and the low expression group contained 27 samples. Demographic data of patients were compared between the high and low expression groups by a two-way ANOVA test. Then, KM metastasis-free survival analysis was conducted using the survival R package. Huvos grades consequent to chemotherapy were recorded in the dataset. Huvos grades 1 and 2 were regarded as poor responses. Conversely, Huvos grades 3 and 4 were regarded as good responses. We compared lncR-C3orf35 and HMGB1 expression between the good and poor response groups.

### 2.8. Tumor Immune Infiltration Analysis

ESTIMATE (Estimation of STromal and Immune cells in MAlignant Tumor tissues using Expression data) is an algorithm used to evaluate the tumor composition by calculating the immune score and stromal score [25]. Using the ESTIMATE R package, the immune and stromal scores were obtained based on the RNA expression data of osteosarcoma samples in the GSE21257 dataset. Then, the scores were compared between the high and low lncR-C3orf35/HMGB1 expression groups. ImmuCellAI is a website tool that estimated the abundance of 24 types of immune cell infiltration in tumor tissue from a gene expression dataset [26]. Normalized expression data of GSE21257 was uploaded to the web portal (http://bioinfo.life.hust.edu.cn/web/ImmuCellAI/). Then, the estimated proportion of immune cell types can was obtained for each tumor sample. For each cell subset, differences in high lncR-C3orf35/HMGB1 and low lncR-C3orf35/HMGB1 expressions were compared.

### 2.9. Cell Culture and qRT-PCR Validation

The normal human osteoblast cell line hFOB 1.19 and osteosarcoma cell line SASJ-2 were purchased from ATCC (USA). The cells were cultured in Dulbecco's Modified Eagle's Medium (DMEM, Gibco, Invitrogen, USA) supplemented with 10% FBS (Gibco, Invitrogen, USA) and grown at 37°C in the presence of 5% CO_2_ air atmosphere.

Total RNA was isolated from cells using TRIzol (Invitrogen) according to the manufacturer's instructions. For the detection of lncR-C3orf35 and HMGB1, the total RNA of each sample was reverse transcribed to cDNA using the PrimeScript RT reagent kit (Takara, Tokyo, Japan). GAPDH served as the internal control. All-in-One™ miRNA real-time quantitative polymerase chain reaction (qRT-PCR) was employed to detect miR-142-3p expression levels using U6 snRNA as the internal control. qRT-PCR was performed with SYBR Premix Ex Taq II (TaKaRa, Tokyo, Japan) on the ABI 7500 thermocycler (Applied Biosystems; Thermo Fisher Scientific, USA). Relative expression was calculated by the 2^-*ΔΔ*Ct^ method, and each experiment was performed in triplicate. ΔΔCt = (Ct_RNA_ − Ct_control_)_tumor_ − (Ct_RNA_ − Ct_control_)_normal_. The experiments were repeated three times. The sequences of real-time PCR primers are listed in Table 2.

### 2.10. Statistical Analysis

R v3.5.3 and GraphPad Prism v8.0 were used for the statistical analysis. Student's *t*-test was used to determine differences between two groups. Any *P* value < 0.05 was considered statistically significant.

## 3. Results

### 3.1. Differentially Expressed lncRNA, mRNA, and miRNA

**Figure 1:**
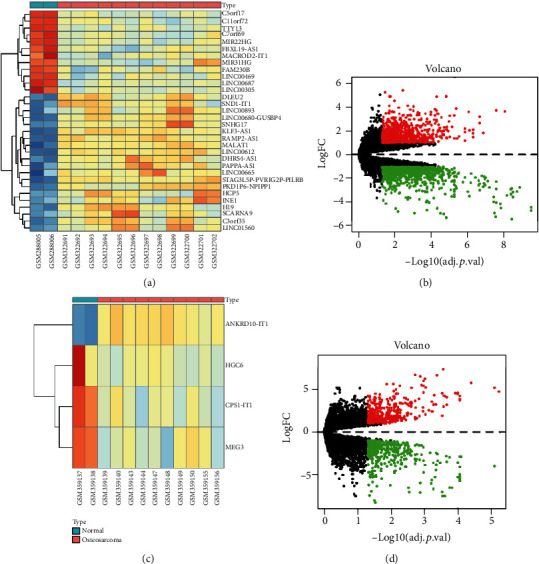
Differentially expressed (DE) lncRNAs and mRNAs in osteosarcoma and normal samples from the GSE14359 and GSE1865 datasets. (a) Heat map of DElncRNAs in GSE12865. (b) Volcano plot of DEGs in GSE12865. (c) Heat map of DElncRNAs in GSE14359. (d) Volcano plot of DEGs in GSE14359. In the heat maps, orange blocks indicate upregulated lncRNAs. Blue blocks indicate downregulated lncRNAs. In the volcano plots, red and green points represent significantly upregulated and downregulated genes, respectively, with ∣logFC | >1 and *p* < 0.05. DEGs: differentially expressed genes.

With the differentially expressed criteria of absolute logFC > 1 and *p* < 0.05, a total of 824 DEmRNAs and 4 DElncRNAs were identified in the GSE143599 dataset (Figure 1). Meanwhile, 1794 DEmRNAs and 32 DElncRNAs were extracted in the GSE12865 dataset. With the union of the two sets, a total of 36 DElncRNAs and 2357 DEmRNAs were obtained. Of these, 21 lncRNAs and 1142 mRNAs were upregulated, while 15 lncRNAs and 1215 mRNAs were downregulated. Additionally, 205 DEmiRNAs were identified in the GSE28423 dataset, of which 101 miRNAs were upregulated and 104 miRNAs were downregulated.

### 3.2. Construction of ceRNA Network

**Figure 2:**
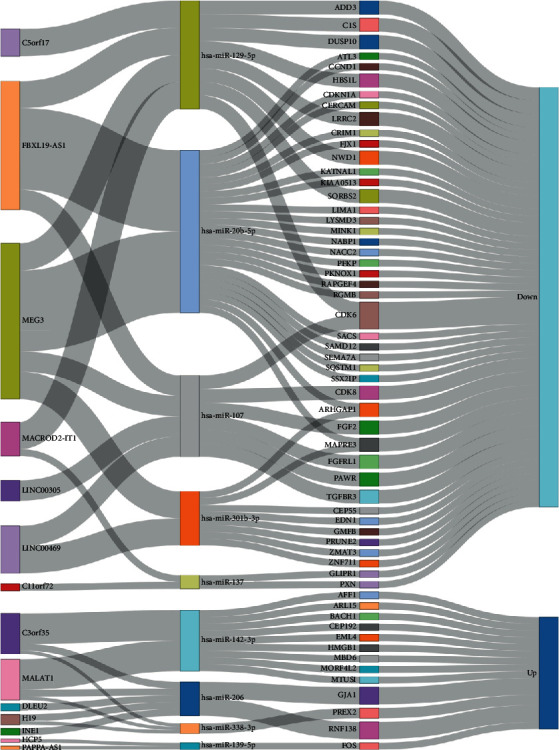
Sankey plot of ceRNA network in osteosarcoma. Blocks in the first, second, and third columns represent lncRNAs, miRNAs, and mRNAs, separately. *Up* in the last column indicates that lncRNAs and mRNAs were upregulated and miRNAs were downregulated. *Down* in the last column indicates that lncRNAs and mRNAs were downregulated and miRNAs were upregulated. Lines indicate the interactions between the two RNAs.

lncRNAs and mRNAs targeted by miRNAs were screened based on the interactions among the DElncRNAs, DEmRNAs, and DEmiRNAs described above. Nine of 205 DEmiRNAs were predicted to interact with 14 of 36 DElncRNAs according to miRcode. Next, 58 mRNAs of 2357 DEmRNAs were screened that targeted 9 DEmiRNAs by searching TargetScan, miRTarBase, and miRDB. Finally, the ceRNA network consisting of 14 lncRNAs, 9 miRNAs, and 58 mRNAs was constructed by Cytoscape software and visualized using the networkD3 R package (Figure 2).

### 3.3. Survival Analysis

**Figure 3:**
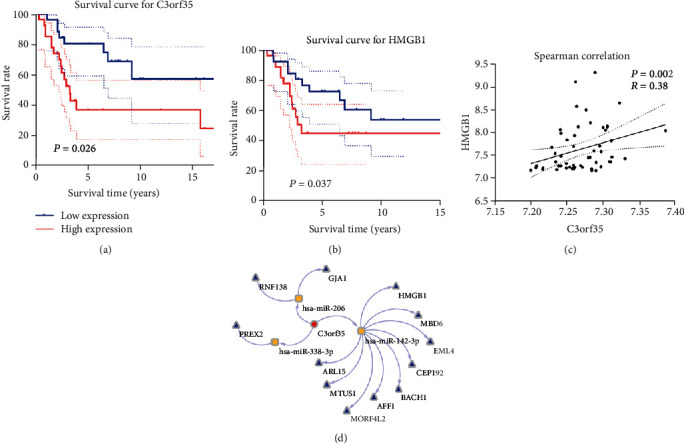
(a) Kaplan-Meier (KM) survival curves for lncR-C3orf35. (b) KM survival curve for HMGB1. (c) Spearman's correlation analysis between HMGB1 and lncR-C3orf35. (d) The ceRNA network mediated by lncR-C3orf35.

Overall survival is considered the ultimate detection standard for the prediction of patient prognosis; hence, screening of survival-associated lncRNAs and mRNAs is important. KM analysis was carried out to explore associations between survival of osteosarcoma patients and DElncRNAs/DEmRNAs included in the ceRNA network. High expressions of both lncR-C3orf35 and HMGB1 were significantly associated with poor overall survival of osteosarcoma patients (Figure 3).

### 3.4. Regression Analysis

Since the data of lncR-C3orf35 and HMGB1 expression was not normally distributed, we performed Spearman's correlation analysis to compare lncR-C3orf35 and HMGB1. The results showed *r* = 0.38 and *p* = 0.002, which indicated that HMGB1 expression was significantly positively correlated with lncR-C3orf35.

### 3.5. GSEA for lncR-C3orf35

**Figure 4:**
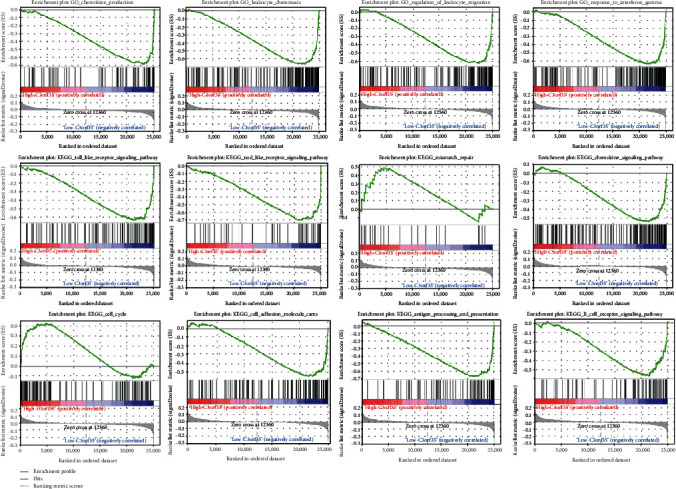
Gene Set Enrichment Analysis for GO and KEGG pathways between high and low lncR-C3orf35 expression groups.

Samples were divided into high and low lncR-C3orf35 expression groups, and analyses of GO biological processes and KEGG GSEA were conducted for the two groups. Forty GO terms were obtained which mainly included terms associated with leukocyte chemotaxis, lymphocyte migration, chemokine production, and response to interferon gamma. Furthermore, KEGG pathways include antigen processing and presentation, B cell receptor signaling, chemokine signaling, Nod-like receptor signaling, and Toll-like receptor (TLR) signaling, as well as cell adhesion molecules (cams), cell cycle, and mismatch repair processes (Figure 4). These biological processes and pathways were associated with cell proliferation, invasion, and immune cell infiltration in the tumor microenvironment.

### 3.6. Clinical Features with lncR-C3orf35/HMGB1

**Figure 5:**
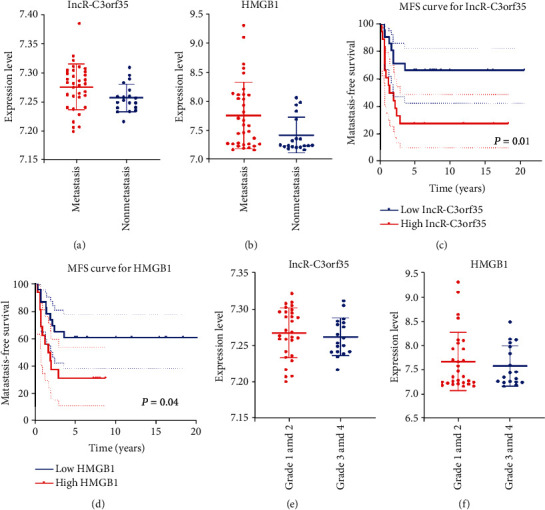
(a) lncR-C3orf35 expression in patients with metastasis and without metastasis. (b) HMGB1 expression in patients with metastasis and without metastasis. (c, d) Metastasis-free survival (MFS) curve for lncR-C3orf35 and HMGB1. (e) lncR-C3orf35 expression in patients with Huvos grades 1 and 2 and patients with Huvos grades 3 and 4. (f) HMGB1 expression in patients with Huvos grades 1 and 2 and patients with Huvos grades 3 and 4.

For further analysis, we investigated correlations between lncR-C3orf35/HMGB1 and clinical features including metastasis and chemotherapy response. The demographic data of patients are displayed in Table 3. There was no difference in the sex and age distribution between the high and low expression groups. Thirty-four of 53 osteosarcoma patients in the GSE21257 dataset presented metastasis at diagnosis or during the follow-up period. lncR-C3orf35 and HMGB1 expression in the metastasis group was significantly higher than that in the nonmetastasis group (lncR-C3orf35: *p* = 0.033; HMGB1: *p* = 0.0075) (Figure 5). Next, samples were classified into the high and low expression groups based on the median expression of either lncR-C3orf35 or HMGB1. KM analysis for metastasis-free survival between high and low lncR-C3orf35/HMGB1 expression groups was performed. The metastasis-free survival rate of the high lncR-C3orf35/HMGB1 expression group was lower than that of the low lncR-C3orf35/HMGB1 expression group (lncR-C3orf35: *p* = 0.01; HMGB1: *p* = 0.04), which indicated that lncR-C3orf35/HMGB1 was correlated with osteosarcoma metastasis. The GSE21257 dataset recorded the Huvos grades of 47 osteosarcoma patients. There are 18 samples in the good response group (Huvos grades 3 and 4) and 29 samples in the poor response group (Huvos grades 1 and 2). lncR-C3orf35 expression in the good response group (7.262 ± 0.026) was similar to that in the poor response group (7.267 ± 0.034, *p* = 0.558). Likewise, there was no significant difference in HMGB1 expression between the good (7.586 ± 0.413) and poor (7.681 ± 0.597, *p* = 0.556) response groups.

### 3.7. Immune Infiltration Analysis

**Figure 6:**
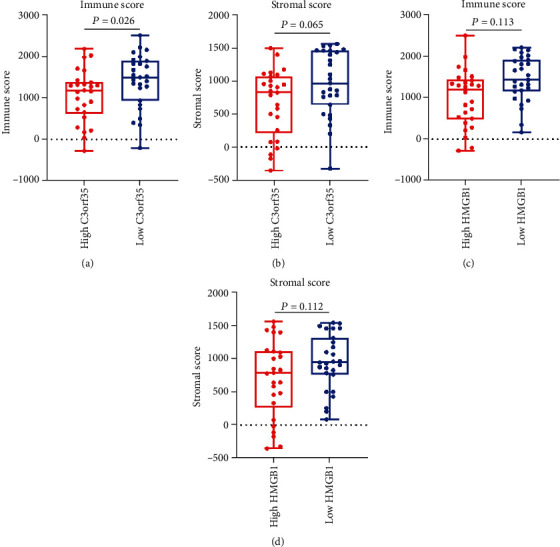
(a, b) Immune scores and stromal scores in the high and low lncR-C3orf35 expression groups. (c, d) Immune scores and stromal scores in the high and low HMGB1 expression groups.

**Figure 7:**
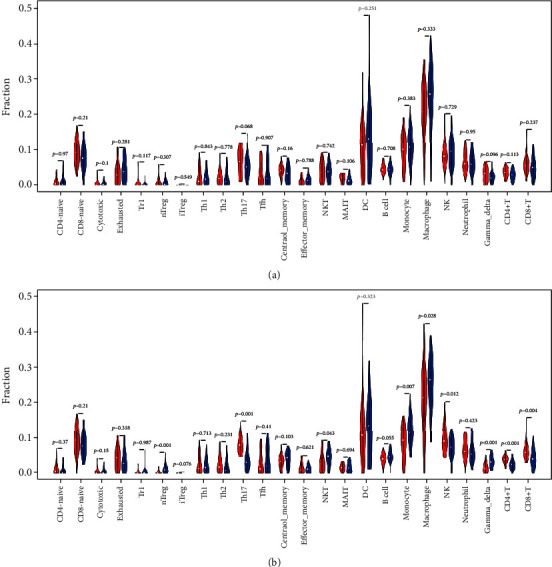
Infiltrating immune cell fractions estimated by ImmuCellAI. (a) Red indicates the high lncR-C3orf35 expression, and blue indicates low lncR-C3orf35 expression. (b) Red indicates high HMGB1 expression, and blue indicates low HMGB1 expression. NK: natural killer; MAIT: mucosal-associated invariant T cell; DC: dendritic cell.

The ESTIMATE algorithm assessing gene signatures was utilized to calculate a stromal score and immune score which represented the proportion of stromal cells and infiltrating immune cells, separately. The stromal score and immune score of each sample in the GSE21257 dataset were obtained using the ESTIMATE R package. The high lncR-C3orf35 expression group presented a significantly lower immune score (1049 ± 626.6 vs. 1400 ± 644.7, *p* = 0.026) than the low lncR-C3orf35 expression group (Figure 6). Similarly, the high HMGB1 expression group presented a lower immune score (996.4 ± 682.8 vs. 1451 ± 549.8, *p* = 0.013) than the low lncR-C3orf35 expression group, while differences in stromal scores between the high and low lncR-C3orf35/HMGB1 expression groups were not significant. Hence, lncR-C3orf35/HMGB1 expression was associated with immune cell infiltration in osteosarcoma. ImmuCellAI was used to evaluate the proportion of 24 immune cell subtypes in tumor tissue basing on gene expression data. Twenty-four subtypes of immune cell proportion of each sample were calculated. The macrophage proportion was lower in the high lncR-C3orf35 expression group (0.207 ± 0.082) than in the low lncR-C3orf35 expression group (0.254 ± 0.089, *p* = 0.042) and was also lower in the high HMGB1 expression group (0.204 ± 0.098) than in the low HMGB1 expression group (0.256 ± 0.071, *p* = 0.032) (Figure 7). The results indicated that macrophage infiltration in osteosarcoma tissue was associated with lncR-C3orf35/HMGB1 expression.

### 3.8. Validation by qRT-PCR

**Figure 8:**
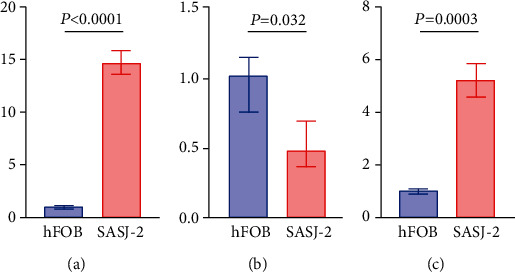
Relative expression levels of lncR-C3orf35, miR-142-3p, and HMGB1 in the osteosarcoma cell line SASJ-2 and normal osteoblast hFOB cells. Every experiment was performed in triplicate.

To validate the bioinformatic analysis results, we utilized qRT-PCR to detect the expression of lncR-C3orf35, miR-142-3p, and HMGB1 in cell lines. lncR-C3orf35 (*p* < 0.0001) and HMGB1 (*p* = 0.0003) were overexpressed in the osteosarcoma cell line SASJ-2 compared to the human osteoblast cell line hFOB 1.19. In addition, miR-142-3p was pressed in low levels in SASJ-2 cells (*p* = 0.03) (Figure 8). The results were consistent with the results described above.

## 4. Discussion

Clinicians face a great challenge when confirming early diagnosis and attempting to predict the prognosis of osteosarcoma patients. Discovering predictive biomarkers and exploring the molecular mechanism of osteosarcoma may provide novel insights for the diagnosis and treatment of osteosarcoma patients. Growing evidence has indicated that dysregulated expression of lncRNAs is associated with carcinogenesis [27]. According to the ceRNA theory, lncRNAs could act as sponges by the binding of base pairs with miRNAs, through which they inhibit miRNA-induced gene silencing. Construction of a ceRNA network contributes to reveal the occurrence and progression of cancer. Systematic analyses of ceRNA networks have been reported in lung cancer [28], gastric cancer [29], and colon cancer [30], but there have been few reports in osteosarcoma.

In this study, we identified 36 lncRNAs, 2357 mRNAs, and 205 miRNAs that were differentially expressed comparing osteosarcoma and normal controls based on RNA microarray data from the GEO database. We constructed a ceRNA regulatory network by utilizing several predictive bioinformatic tools. Through KM analysis, the lncR-C3orf35/HMGB1 pair was found to be associated with the overall survival of osteosarcoma patients. GSEA analysis was performed and revealed that lncR-C3orf35 was involved in cell adhesion, regulation of the cell cycle, and immune cell infiltration. Next, we found that lncR-C3orf35 and HMGB1 expression was correlated with osteosarcoma metastasis. Patients with high expression of lncR-C3orf35/HMGB1 presented lower metastasis-free survival. Using the ESTIMATE algorithm, immune scores of the high lncR-C3orf35/HMGB1 expression group were significantly lower than those of the low expression group, which suggests that immune cell infiltration differed between these two groups. We calculated the proportion of immune cell subtypes for each sample utilizing the ImmuCellAI online tool and found that the macrophage proportion in the high lncR-C3orf35/HMGB1 expression group was significantly lower than that in the low expression group. Moreover, the expression of lncR-C3orf35, miR-142-3p, and HMGB1 was validated in cell lines by qRT-PCR.

LncRbase (http://bicresources.jcbose.ac.in/zhumur/lncrbase/index.html) showed that lncR-C3orf35 is located in chr3:3744065-37476988 and has a length of 574 bases. There have been no reports on the association between lncR-C3orf35 and cancer. Our study showed that lncR-C3orf35 was overexpressed in osteosarcoma tissue and cells. In addition, upregulation of lncR-C3orf35 was found to be associated with poor overall survival and metastasis-free survival, which indicated that lncR-C3orf35 was a potential biomarker of osteosarcoma development.

The GSEA KEGG analysis identified an enriched TLR pathway, which could bind HMGB1 and activate a series of inflammatory responses [31]. Our results showed that lncR-C3orf35/HMGB1 expression was correlated with a low immune score and low macrophage infiltration. Previous studies have reported that a high macrophage proportion in osteosarcoma tissue was associated with better prognosis and inhibition of metastasis [32, 33]. Macrophages are a crucial member of the innate immune system and are also the most abundant immune cell in the tumor microenvironment [34]. Tumor-associated macrophages differentiate into M1-type and M2-type macrophages under various cytokine stimulation. M1 macrophages mediate inflammation and kill tumor cells, while M2 macrophages promote tumor growth and induce immune suppression. The balance of M1/M2 is closely associated with tumor metastasis [35]. HMGB1 has been reported to induce tumor-associated macrophage polarizing to M2 macrophages through the NF-*κ*B signaling pathway [36]. Hence, the lncR-C3orf35/HMGB1 axis may affect the polarization of macrophages, which promotes osteosarcoma metastasis.

HMGB1 is a highly conserved protein with 215 amino acids and 3 structural domains. It was initially regarded as a nuclear protein regulating transcription. HMGB1 has been shown to bind TLRs and RAGEs, through which it induces the secretion of various proinflammatory cytokines and triggers a series of inflammatory responses [37]. Under HMGB1 stimulation, angiogenesis and immune inhibition promote tumor metastasis [38]. Conversely, high levels of HMGB1 in osteosarcoma may induce autophagy of osteosarcoma cells, which contributes to resistance to cisplatin, doxorubicin, and methotrexate treatment [39]. Nonetheless, our study did not identify any correlation between HMGB1 expression and the chemotherapy response due to the small sample size. Extracellular HMGB1 protein results in higher osteosarcoma cell proliferation, migration, and osteogenic differentiation [40]. Our study showed that high HMGB1 expression was associated with metastasis and immune cell infiltration of osteosarcoma, which was consistent with previous studies. In addition, we propose that HMGB1 may mediate the influence of lncR-C3orf35 on osteosarcoma prognosis through a ceRNA mechanism.

Xu et al. [41] reported that miR-142-3p was downregulated in osteosarcoma tissue and cell lines. Several target mRNAs of miR-142-3p have been proposed including HMGA1, HMGB1, and Rac1 [42]. In hepatocellular cancer, miR-142-3p was shown to bind the 3′-UTR region of HMGB1 and resulted in the inhibition of proliferation and invasion of tumor cells [43]. Moreover, miR-142-3p has the potential to predict prognosis of colorectal cancer [44] and renal carcinoma [45] patients. In our study, miR-142-3p could interact with lncR-C3orf35 and HMGB1 based on predictive bioinformatic tools. We further confirmed differences in gene expression by microarray data and qRT-PCR, which supported the expression of the lncR-C3orf35/miR142-3p/HMGB1 axis in osteosarcoma. Further studies are needed to explore the biological functions of the lncR-C3orf35/miR142-3p/HMGB1 axis in osteosarcoma.

In conclusion, we identified dysregulated lncRNAs, miRNAs, and mRNAs in osteosarcoma and constructed a ceRNA regulatory network, which contribute to a better understanding of osteosarcoma progression. Several genes in the network were shown to be dysregulated and to function as oncogenes or tumor suppressors in osteosarcoma, such as HMGB1 and miR-142-3p. lncR-C3orf35 and HMGB1 were associated with poor prognosis of osteosarcoma patients. We propose that lncR-C3orf35 may upregulate HMGB1 by sponging miR-142-3p. Moreover, lncR-C3orf35 and HMGB1 may affect immune cell infiltration and tumor metastasis. Thus, our study supports lncR-C3orf35 as a new biomarker of osteosarcoma carcinogenesis and as a potential therapeutic target.

## Figures and Tables

**Table 1:** Differentially expressed RNAs in GEO datasets.

Accession numbers	Microarray platform	Tumor samples	Normal samples	DElncRNAs		DEmRNAs		DEmiRNAs	
				Up	Down	Up	Down	Up	Down
GSE14359	GPL96	10	2	1	3	414	410	—	—
GSE12865	GPL6244	12	2	20	12	852	942	—	—
GSE28423	GPL8277	19	4	—	—	—	—	101	104

Abbreviations: up: upregulated; down: downregulated; DE: differentially expressed.

**Table 2:** Sequences of qRT-PCR primers.

Symbols	Sequences
C3orf35	F: 5′-AAGAGGTTATTGTGCGCCCG-3′
C3orf35	R: 5′-ATTAGCCCGCCTTCCTCTGT-3′
miR-142-3p	F: 5′-CAGCTGGGTGTAGTGTTTCCTACTT-3′
miR-142-3p	R: 5′-ACGCAGGGTCCGAGGTATTC-3′
HMGB1	F: 5′-GGTCATCACACACGGAGCTG-3′
HMGB1	R: 5′-AACGGGTCGTGGAATGCAAA-3′
ACTB	F: 5′-CATGTACGTTGCTATCCAGGC-3′
ACTB	R: 5′-CTCCTTAATGTCACGCACGAT-3′
U6	F: 5′-CGCTTCGGCAGCACATATAC-3′
U6	R: 5′-AAAATATGGAACGCTTCACGA-3′

Abbreviations: F: forward primer; R: reverse primer.

**Table 3:** Demographics of patients in GSE21257.

Characteristics		Low C3orf35	High C3orf35	Significance	Low HMGB1	High HMGB1	Significance
Sex	Male	15	19	NS	16	18	NS
	Female	12	7		11	8	

Age	<10	3	1	NS	2	2	NS
	10~15	5	11		8	8	
	15~20	13	9		12	10	
	>20	6	5		5	6	
	Median	17.0	15.0	NS	16.7	16.0	NS

NS: no significant difference.

## Data Availability

All the datasets used for this study can be found in GEO: https://www.ncbi.nlm.nih.gov/geo/query/acc.cgi?acc=GSE14359, https://www.ncbi.nlm.nih.gov/geo/query/acc.cgi?acc=GSE12865, https://www.ncbi.nlm.nih.gov/geo/query/acc.cgi?acc=GSE28423, and https://www.ncbi.nlm.nih.gov/geo/query/acc.cgi?acc=GSE21257.
